# Translation, Reliability and Validity of the Spanish Version of the Modified New Mobility Score (NMS-ES)

**DOI:** 10.3390/ijerph18020723

**Published:** 2021-01-15

**Authors:** Rafael Prieto-Moreno, Patrocinio Ariza-Vega, Mariana Ortiz-Piña, Maureen C. Ashe, Dulce Romero-Ayuso, Morten Tange Kristensen

**Affiliations:** 1 PA-HELP “Physical Activity for HEaLth Promotion” Research Group, Department of Physical Education and Sport, University of Granada, 18011 Granada, Spain; rafaprieto58@hotmail.com; 2 Department of Physiotherapy, University of Granada, 18016 Granada, Spain; marianaorpi@gmail.com (M.O.-P.); dulceromero@ugr.es (D.R.-A.); 3 Biohealth Research Institute, Physical Medicine and Rehabilitation Service, Virgen de las Nieves University Hospital, 18016 Granada, Spain; 4 Department of Family Practice, The University of British Columbia, Vancouver, BC V6T 1Z3, Canada; maureen.ashe@ubc.ca; 5 Centre for Hip Health and Mobility, Vancouver, BC V5Z 1M9, Canada; 6 Physical Medicine and Rehabilitation Research—Copenhagen (PMR-C), Departments of Physiotherapy and Orthopaedic Surgery, Amager-Hvidovre Hospital, University of Copenhagen, 2650 Hvidovre, Denmark; mortentange@hotmail.com; 7 Institute of Clinical Medicine, University of Copenhagen, 2200 Nørrebro, Denmark

**Keywords:** multidisciplinary/interdisciplinary rehabilitation, geriatric rehabilitation, trauma rehabilitation, occupational therapy, physical therapy

## Abstract

The New Mobility Score (NMS) is an easy to administer self-report measure of functional ability, and is used worldwide as a hip fracture (HF) score, but a Spanish version does not exist. The aim of the study is to translate NMS into Spanish, and to measure its inter-rater reliability, internal consistency, and concurrent validity in a sample of Spanish speaking patients with HF. A reliability and validity study with a sample of 60 adults, 65 years or older (46 women and 14 men; mean age 81.7 years) with a hip fracture admitted consecutively to the acute trauma service of the Health Campus Hospital of Granada. The participants were interviewed during the first week after surgery by an occupational therapist or a physiotherapist. The statistical test used for analysis were: Cronbach’s α coefficient, McNemar–Bowker test, Bland–Altman plot, Spearman´s Rho, and Mann–Whitney U test. The Cronbach’s α coefficient was 0.90. No inter-rater systematic differences were found. We noted significant associations between the Spanish Version of the Modified New Mobility Score (NMS-ES) and selected health outcomes: Age, cognition, pre-fracture function, and basic mobility. The NMS-ES is a reliable and valid instrument to assess pre-injury functional levels for patients with HF in Spanish speaking countries.

## 1. Introduction

One of the most significant consequences of hip fractures for many older adults is the persistent loss of function [1]. The etiology of functional loss post-hip fracture is multifactorial, and well documented in the literature, including factors such as: Age, cognitive impairment [2], comorbidities [3], low prefracture functional level [2,4], delay of hip fracture surgery [5], and late mobilization and rehabilitation after surgery [6]. The Barthel Index [7], Functional Independence Measure (FIM) [8], Katz Index of Independence Measure [9], and the New Mobility Score (NMS) [10] are frequently used scales in clinical practice globally to assess and monitor function. Of these, the Barthel, FIM, and Katz Index have been translated and validated into several languages, including Spanish [11,12,13]. Even though Spanish is the second most spoken language in the world, the NMS has to our knowledge not been translated into Spanish yet, which would be beneficial for clinical care and in the research setting [3,14], due to the fast use of the NMS-ES when compared with others assessment instruments and the limited time available to health care providers so as to care for patients with hip fracture in acute care settings.

The NMS was initially developed by Parker and Palmer [10], and later modified by Kristensen and Kehlet [15]. It is a valid predictor of long-term mortality [10,16], short-term mobility outcomes [15,17], and hospital discharge destination [4,15]. The NMS has three self-reported questions related to pre-fracture walking ability that can be obtained within a few minutes. It is reliable [18], and can easily measure pre-fracture function that is understandable for patients and for caregivers of patients with cognitive impairment. Thus, the NMS can be used to measure pre-fracture function for all patients with hip fracture. This is a crucial factor, as up to one-third of all those patients with hip fractures have moderate or severe cognitive impairment [19].

The NMS has the potential to support research, registries, and hip fracture clinical care, which nowadays are focused on the development of new ways of treatment, apart from the traditional rehabilitation protocol in the balance and walking ability during the hospital stay [1,2,20,21]. It provides a self-reported pre-fracture functional baseline for clinicians to monitor the recovery process [10]. Furthermore, the NMS is gaining popularity as an instrument in clinical research conducted across different countries such as the United States [22], Denmark [16], and Ireland, where it is included in the Irish Hip Fracture Database [17,23]. Despite its many benefits, NMS has not been adapted for Spain, a country with significant adjusted rates of hip fracture: 722/100,000 for women and 284/100,000 for men [24].

Therefore, the aim of this study is to translate the modified English version of the NMS into Spanish (NMS-ES), and to measure its reliability and validity for health professionals in an acute orthopedic unit. Adapting and evaluating the NMS-ES would provide clinicians and patients of different Spanish speaking countries an easy to use, reliable, and valid clinical instrument to support the recovery process, and advance research and clinical practice.

## 2. Materials and Methods

### 2.1. New Mobility Score

The NMS consists of three questions to measure walking mobility across daily life activities. If possible, the NMS is completed by the person being evaluated, or by a caregiver for people with cognitive impairment [18]. The NMS asks for a self-report (on a four point ordinal scale) of their ability to complete: (i) Indoor walking (e.g., in a house); (ii) outdoor walking; and (iii) walking during shopping [18]. Each question is scored from 0 to 3 points based on the person’s self-reported ability [15]: Zero points indicates the person is not able to complete the task; for 1 point, the person requires help from another person; 2 points means the person completed the task with a walking aid; and 3 points means the person completed the task independently without a walking aid [15,18]. Total scores range from 0–9 points; 6 points or less indicate functional impairment; and above 6 points signify a high level of prefracture function in patients with hip fracture [4]. In addition, some research has shown that a score ≥ 7 indicates the patient may be able to return home from acute unit care directly and not need inpatient rehabilitation [4].

### 2.2. Translation of the NMS-ES

We translated the English version of the modified NMS manual following the recommendations of Ramada-Rodilla and colleagues [25]. Two occupational therapists (OT), unfamiliar with the NMS, independently translated the modified English version [15], including frequently asked questions (FAQ) into Spanish, after approval for translation was given by one of the authors of the modified NMS (Morten T. Kristensen). Modifications were made in order to be consistent with Spanish culture and language while preserving the original meaning. A physiotherapist (PT), also unfamiliar with the NMS, created a third version by merging the two previous versions. Following this, a committee of two OTs, a PT, and a physician reviewed the merged version, and finalized a fourth version. The committee’s aim was to correct possible Spanish culture and language errors or misunderstandings in the translated version. The final version was back translated into English by a native English speaker (unfamiliar with the NMS). The back translated English version was reviewed and approved by an author of the modified NMS (Morten T. Kristensen). We made minor changes to the NMS-ES based on this feedback. Please see the final version of the NMS-ES in Appendix A. All people involved in the translation process approved the final version.

### 2.3. Reliability

We included 60 patients 65 years and older with hip fractures admitted consecutively to the acute trauma service of the Health Campus Hospital of Granada between January 2017 and March 2017. The study was approved by the ethics committee of the Research Center of Granada (cBI-cni.Nana 28 February 2017) and was carried out according to the guidelines established by the Helsinki Declaration and Law 14/2007 on Biomedical Research. All patients or their caregivers (for patients with cognitive impairment), signed an informed consent form before participating in the study.

In the present study, patients and their caregivers were interviewed by an OT and PT during the first week after surgery. Each patient or his/her caregiver (in case of patients with severe cognitive impairment) were interviewed and asked for NMS-ES questions by one of the therapists. The therapists exchanged the role of interviewer, so each one asked the questions to 30 patients or caregivers. Both therapists completed the scale, blinded to each other’s rating until the end of study, using standard procedures [26]. Patients without cognitive impairment and caregivers of patients with severe cognitive impairment were requested to provide a response based on patients’ ability to perform the tasks considered in the NMS during the week prior to the hip fracture. This method was used to determine the inter-rater reliability for the NMS-ES.

To determine the concurrent validity, we collected the following data during the hospital interview: Age, gender, highest level of education, place of residence before fracture (own home, with family, nursing home, or other), change of residence after hospital discharge (yes or no), previous falls during the last year (yes or no), support at hospital discharge (formal caregiver or informal caregiver), pre-fracture functional level (assessed by the Functional Independence Measure, [FIM, 18 (minimum level of independence) to 126 (maximum level of independence)]) [8], cognitive status [(using the Short Portable Mental Status Questionnaire test (SPMSQ) (SPMSQ test 0–10 points)] coded as: <3 points = no cognitive impairment; 3–4 points = mild cognitive impairment; 5–7 points = moderate cognitive impairment; and >8 points = severe cognitive impairment] [27,28], and basic mobility [assessed by the Cumulated Ambulation Score, Spanish version (CAS-E)] [29]. We also collected the following information from patients´ medical record: Weight and height (to calculate the Body Mass Index; BMI), type of hip fracture, and type of surgery.

### 2.4. Statistical Analysis

We calculated the sample size following the suggestion to include at least five participants per questionnaire item [30], and the recommendations to include a minimum of 50 participants for reliability estimates [31]. The NMS has three items with four options for each item, therefore we included a total of 60 patients in the present study.

We calculated mean (standard deviation; SD) or median (interquartile range; IQR), depending on the distribution of data as indicated by the Kolmogorov–Smirnov Test. Cronbach´s Alpha test was used to determine internal consistency of the NMS-ES. Using this test, results range from 0 to 1, with values closer to 1 indicating greater consistency [32]. For the measurement of the inter-rater reliability of the NMS-ES, Bland-Altman analysis was carried out so as to analyze scoring differences between the two raters measurements [33,34]. We assessed systematic inter-rater bias using the McNemar–Bowker test.

For testing concurrent validity, we use Spearman´s Rho to explore the association between NMS-ES and selected variables based on the literature [4,15]: Age, cognition, the pre-fracture FIM, the CAS-E after surgery. Mann–Whitney U test was used to compare the results of the NMS-ES between patients according to their pre-fracture residential status (own home versus nursing or relative home). For all analyses, we used IBM SPSS Statistics Version 25.0 (IBM Corp., Armonk, NY, USA).

## 3. Results

### 3.1. Translating the NMS-ES

We translated the NMS-ES as described in the Methods. The main differences between versions one and two (developed independently by two OTs) were grammar-related, and a change in the shopping activity: “walking when the person goes shopping” vs. “walking during shopping time”). The third version of NMS-ES, (developed by a PT) decided on the term “walking during shopping”, and adopted the use of the passive voice to describe the scoring process. The fourth (and final) version of the NMS-ES developed by the committee changed the term “technical aids or aids” (used in the three previous versions) to “supportive devices”, a term more appropriate to use in Spanish culture and language. The back translated version resulted in some formatting changes, and minor changes in wording, such as “punctuation” for “scoring”, “punctuate” for “record”, and “due to the fact that” for “although”.

### 3.2. Reliability and Validity of the NMS-ES

**Figure 1:**
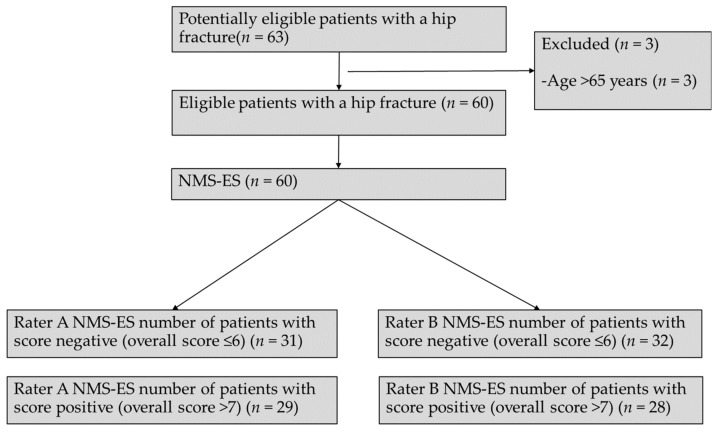
Flow diagram of the NMS-ES (Spanish version of the New Mobility Score).

The flow diagram of the participants in the development of the NMS-ES is shown in Figure 1.

The sociodemographic and clinical data of the 60 patients with hip fracture are provided in Table 1. Patients or their caregivers were asked for their pre-fracture NMS-ES between the second and sixth day after surgery. It took approximately 3–5 mins to complete the NMS-ES. Rater A classified 31 patients of 60 with 6 points or less and 29 patients with an overall bigger than 6 points, meanwhile rater B assigned 6 points or less for 32 patients and over 6 points for 28 patients.

### 3.3. Reliability

The internal consistency of the total NMS-ES presents a Cronbach’s α coefficient of 0.90, and with the corresponding data of each item shown in Table 2. The correlations between the three items were a mean of 0.81 with a minimum of 0.77 and a maximum of 0.86. The scores by the two raters differed in one of the 60 patients, but the difference was only 1 point.

**Figure 2:**
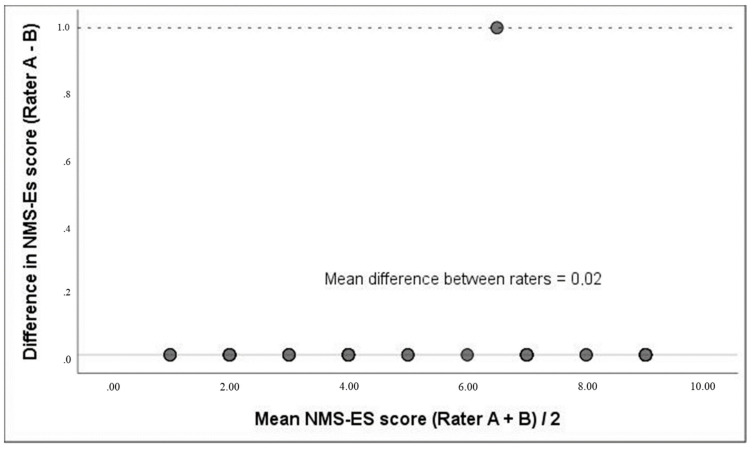
Bland–Altman plot of an occupational therapist (rater A) and a physiotherapist (rater B) scores for the Spanish version of the New Mobility Score (NMS-ES).

The scores by two raters only differed in one point in an only patient, as illustrated in the Bland–Altman plot (Figure 2). The mean of differences between these two measurements was 0.02 (95% confidence interval (CI): (−0.23–0.27). There was no systematic inter-rater bias for the overall NMS-ES.

### 3.4. Validity

Correlations of NMS-ES with: Age was r = −0.42 (*p* = 0.001); cognitive status (SPMSQ Test) was r= −0.71 (*p* > 0.001); pre-fracture FIM was r = 0.64 (*p* < 0.001); CAS-E assessed between day 2 and 6 post-surgery was r = 0.70 (*p* < 0.001). The mean (SD) of the NMS-ES was 3.33 (1.80) for patients from nursing homes or relative’s homes vs. 6.48 (2.60) for patients living at home (*p* < 0.001).

## 4. Discussion

The modified English version of the NMS was successfully translated into Spanish, and was a reliable and valid instrument to evaluate the pre-fracture functional status of patients who sustained a hip fracture. In addition, excellent internal consistency, almost perfect agreement, moderate correlation with age, and strong correlation with cognitive status and functional level was observed.

The NMS-ES demonstrated excellent internal consistency (Cronbach’s α = 0.90), even though the scale is only composed of three items; short scales usually decrease the value of α coefficient [32]. This Cronbach’s α result is considered as the minimum value for clinical application by Bland and Altman [35]. According to Tavakol and Dennick, who consider a Cronbach’s α = 0.7 as acceptable [32], the NMS-ES has a better than acceptable internal consistency. The good agreement between raters shown in the Bland–Altman plot [33,34], with only one difference is a result consistent with that previously established for the NMS [intraclass correlation coefficient (ICC) = 98% (95 CI 0.96–0.99)] [18]. Raters of the present study were a PT and OT, providing support for the use of the instrument by different health care providers. The use of scales that can be administered by different health care providers facilitate the interdisciplinary work in clinical practice. According to its excellent internal consistency and inter-rater reliability, NMS-ES could be considered as a useful instrument in acute care settings where the large number of patients and limited staff resources usually result in low priority for assessing patients’ function.

Age and cognitive impairment were moderately and strongly negatively correlated with the NMS-ES, respectively [36], which confirms previous findings [15]. The results are particularly relevant if we consider possible difficulties assessing patients with cognitive impairment. One of the advantages of the NMS is the ability for caregivers to provide relevant information in those cases when the patient is not able to do it due to its cognitive impairment. This feature extends the instrument to include all patients with hip fracture, including patients with cognitive impairment, a group frequently excluded in research for this clinical area [37]. Thus, beyond the clinical utility of the NMS-ES, it can provide valuable insights for research. These results provide further support for the NMS, in general, as an important instrument to measure patients’ pre-fracture functional level. We need to rely on patients’ and caregivers’ perception of pre-fracture function to provide context for goal setting and overall management.

The association of the NMS-ES with the FIM was considered, according to previous studies, as strong [36]. Both scales have shown excellent reliability and almost perfect agreement and good validity [8,18]. However, the NMS was specifically designed for older people with hip fractures and it requires much less time to complete compared with the FIM; although it also provides less information. Nevertheless, because of limited time, the clinicians who work in acute care settings request short scales that can be easily incorporated into practice to develop appropriate rehabilitation strategies. Further, short hospital stays also supports the use of quick and easy to use instruments to ascertain important information such as pre-fracture function.

The NMS-ES was also strongly correlated with the CAS-E [34]. These findings are consistent with the study by Kristensen et al. [4], that reported patients with a low pre-fracture NMS would be 18 times more likely not to regain independence in basic mobility, assessed by the CAS, when discharged from an acute orthopedic ward. Corresponding findings were reported by Hulsbæk et al. [38], and Fitzgerald et al. [17]. The relation of the NMS as a predictor of the basic mobility of patients after surgery supports the recommendation to include the NMS-ES in the assessment protocol for patients admitted to hospital with hip fractures. Further, the association between the NMS-ES and the place of residence showed that people who lived in their own home before the fracture, as expected, presented higher NMS-ES scores than those not living in their own home, due to these patients usually being less involved in carrying out their activities of daily living by having professionals or relatives at their disposal.

These results proved the concurrent validity of the NMS-ES as an assessment tool to measure the pre-injury functional level in patients with a hip fracture.

### Study Strengths and Limitations

This study has many strengths to support clinical practice and research. First, we provided a comprehensive approach to the translation of the NMS into Spanish according to international recommendations [23]. Second, to test some psychometric properties of the NMS-ES, we enrolled a representative sample of patients with hip fracture across different levels of cognitive status, types of fracture, and places of residence [39]. Third, we confirmed the results of the NMS-ES with previously validated scales such as the FIM [8] and the CAS-E [27]. However, we also acknowledge some limitations regarding the interrater estimates. Despite of the fact that the two raters were blinded to each other’s NMS scoring, they were both present when the instrument explanation were provided to patients and their caregivers. This may explain the almost 100% interrater agreement. Nevertheless, our results are consistent with the only previous inter-rater reliability study of the NMS [18]. Finally, the NMS-ES has been translated into the European Spanish language and validated in a European Spanish population. A cross-cultural adaption of the NMS-ES is required for its use in other Spanish speaking countries.

## 5. Conclusions

The NMS-ES is a reliable and valid outcome measure to assess the pre-fracture functional status of older patients with hip fracture in Spain. We confirm the NMS-ES as an easy to use and quick to complete score that can be used for all patients with hip fracture, with information provided by the caregivers for those patients with cognitive impairment. Furthermore, it is reliable when used by OTs and PTs, important for consistency within team-based assessment of patients with hip fracture. Overall, the NMS-ES has strong potential as an important instrument for clinical assessment and monitoring pre-fracture function of patients with hip fracture in Spain, but also for follow-up assessments to monitor the level of recovery after fracture.

## Figures and Tables

**Table 1:** Sociodemographic and clinical data of 60 patients with hip fracture.

Variable	*N* = 60
Age, years: Mean (standard deviation), minimum–maximum	81.7 (6.8), 65–96
Gender *n* (%)	
Women	46(77)
Men	14 (23)
Body Mass Index kg/ m2 Classification *n* (%)	
Underweight (<18.5)	1 (2)
Normal (18.5–24.9)	18 (30)
Overweight (≥25)	41 (68)
Educational level *n* (%)	
Cannot read and write	16 (27)
Can read and write	25 (42)
Primary school	13 (22)
High school	3 (5)
College (University)	3 (5)
Pre-fracture Functional Independence Measure: median (IQR)	100.5 (79–123.8)
Cognitive Status (SPMSQ test, 0–11 points) *n* (%)	
No cognitive impairment (0–3 points)	27 (45)
Mild cognitive impairment (3–4 points)	15 (25)
Moderated cognitive impairment (5–7 points)	9 (15)
Severe cognitive impairment (8–11 points)	9 (15)
Type of fracture *n* (%)	
Cervical Femoral (Intracapsular)	40 (67)
Trochanteric (Extracapsular)	20 (33)
Type of surgery *n* (%)	
Prosthesis	28 (47)
Intramedullary hip screw	32 (53)
Falls in the previous year *n* (%)	
Yes	18 (30)
No	42 (70)
Pre-fracture residence *n* (%)	
Own home	45 (75)
Nursing or relative´s home	15 (25)
Change of residence at hospital discharge *n* (%)	
Yes	17 (28)
No	43 (72)
Support at hospital discharge *n* (%)	
Formal caregiver	14 (23)
Informal caregiver (relative or friend)	46 (77)
Post-surgery Cumulated Ambulation Score assessed during the first week (between day 2 and 6 from surgery): median (IQR)	3 (2–5)

Values are presented as; number of patients (%), mean (standard deviation), minimum–maximum or as median (IQR) as appropriate.

**Table 2:** Internal consistency (Cronbach’s α) of the Spanish version of the New Mobility Score.

Item	α Coefficient if Item Deleted
Indoor walking	0.92
Outdoor walking	0.79
Walking during shopping	0.84
Total	0.90

## Data Availability

The data presented in this study are available on request from the corresponding author. The data are not publicly available due to the privacy of the participants personal data, detailed in the informed consent.

## Appendix A. Spanish Version of the Modified New Mobility Score (NMS-ES)

**Table A1:** Spanish Version of the Modified New Mobility Score (NMS-ES).

Nuevo Test de Movilidad (NMS-ES, 0–9 Puntos)				
Movilidad	Sin dificultad Y sin ningún dispositivo de apoyo	Con un dispositivo de apoyo para caminar	Con ayuda de otra persona	De ningún modo
Capaz de moverse por la casa (Caminar dentro)	3	2	1	0
Capaz de salir de la casa (Caminar fuera)	3	2	1	0
Capaz de ir de compras (Caminar cuando va de compras)	3	2	1	0

Spanish version by Prieto-Moreno R et al., 2019, after modified English version by Kristensen MT January 2010, from Parker and Palmer [10]. J Bone Joint Surg 1993; 75: 797–9 approved by Dr. Parker, and published in Kristensen and Kehlet. Danish Medical Journal 2012; 59: A4447 [15].

El Nuevo Test de Movilidad (NMS-S) fue diseñado en su origen para pacientes con fractura de cadera [10], pero puede usarse para otros grupos diagnósticos con problemas de movilidad [40,41]. El NMS valora la marcha; dentro de la casa, fuera de la casa y al ir de compras. Se asignan de 0 a 3 puntos para cada función, resultando una puntuación total de 0 a 9 puntos. Aunque no ha sido evaluado formalmente, una mejora de un punto en el NMS indica un cambio clínicamente relevante. La excelente fiabilidad Inter evaluador del NMS en pacientes con fracturas de cadera [18], ha sido probada, así como su elevada utilidad predictiva sobre la mortalidad y otras consecuencias de la fractura de cadera [4,10,15,16,17,38]. El NMS puede utilizarse para la evaluación del cambio y evolución de la movilidad tras una fractura de cadera [3,42,43].

**Manual para la puntuación del Nuevo Test de Movilidad (NMS-ES):**

Si el paciente utiliza ocasionalmente algún dispositivo de apoyo o una silla de ruedas para las actividades descritas, se puntúa con el nivel más bajo de función.

Al evaluar, por ejemplo, el nivel funcional previo, se le pide al paciente que recuerde su funcionalidad en la semana previa a la fractura de cadera. Muchos pacientes tienden a describir su nivel funcional previo refiriéndose a meses o años atrás, por lo que hay que confirmar su respuesta, por ejemplo, el caminar fuera de casa, se les pregunta cuándo fue la última vez que estuvieron fuera de casa o que bajaron las escaleras.

A una persona que usa el coche como medio de transporte para ir a comprar y utiliza un bastón mientras compra, se le asignan 2 puntos en ir de compras.

En personas con deterioro cognitivo, con el fin de garantizar la exactitud, la información sobre la movilidad del paciente será obtenida a través de los cuidadores del paciente en casa o en la residencia.

A una persona, que por ejemplo usa silla de ruedas fuera de la casa o para ir de compras se le asignan 0 puntos tanto para caminar fuera como para ir de compras.

A una persona que no usa dispositivos de apoyo para moverse dentro de la casa, pero que se apoya en muebles, marcos de las puertas o similares, se le asignan 2 puntos en la actividad de caminar dentro.

**Dispositivo de apoyo terapéutico para caminar:**

Dentro de casa ______________ Fuera de casa_______________ Al ir de Compras___________

**Resultados:**

Dentro de casa (0–3) ___ Fuera de casa (0–3) ____ Al ir de Compras (0–3) ____ Total (0–9) ____

El NMS es también conocido como el” Test de Movilidad de Parker” aunque fue desarrollado conjuntamente con el Dr Palmer. La presente versión del NMS debería ser citada como:” La versión modificada y fiable del Nuevo test de Movilidad” [10,15,18].

## References

[1] Dyer S.M., Crotty M., Fairhall N., Magaziner J., Beaupre L.A., Cameron I.D., Sherrington C. (2016). A critical review of the long-term disability outcomes following hip fracture. BMC Geriatr..

[2] Tseng M.-Y., Shyu Y.-I.L., Liang J. (2012). Functional Recovery of Older Hip-Fracture Patients After Interdisciplinary Intervention Follows Three Distinct Trajectories. Gerontologist.

[3] González-Zabaleta J., Pita-Fernandez S., Seoane-Pillado T., López-Calviño B., Gonzalez-Zabaleta J.L. (2016). Comorbidity as a predictor of mortality and mobility after hip fracture: Mortality and mobility after hip fracture. Geriatr. Gerontol. Int..

[4] Kristensen M.T., Foss N.B., Ekdahl C., Kehlet H. (2010). Prefracture functional level evaluated by the New Mobility Score predicts in-hospital outcome after hip fracture surgery. Acta Orthop..

[5] Klestil T., Röder C., Stotter C., Winkler B., Nehrer S., Lutz M., Klerings I., Wagner G., Gartlehner G., Nussbaumer-Streit B. (2018). Impact of timing of surgery in elderly hip fracture patients: A systematic review and meta-analysis. Sci. Rep..

[6] Morri M., Forni C., Marchioni M., Bonetti E., Marseglia F., Cotti A. (2018). Which factors are independent predictors of early recovery of mobility in the older adults’ population after hip fracture? A cohort prognostic study. Arch. Orthop. Trauma Surg..

[7] Collin C., Wade D.T., Davies S., Horne V. (1988). The Barthel ADL Index: A reliability study. Int. Disabil. Stud..

[8] Ottenbacher K.J., Hsu Y., Granger C.V., Fiedler R.C. (1996). The reliability of the functional independence measure: A quantitative review. Arch. Phys. Med. Rehabil..

[9] Katz S. (1963). Studies of Illness in the Aged: The Index of ADL: A Standardized Measure of Biological and Psychosocial Function. JAMA.

[10] Parker M., Palmer C. (1993). A new mobility score for predicting mortality after hip fracture. J. Bone Joint Surg. Br..

[11] Martínez-Martín P., Fernández-Mayoralas G., Frades-Payo B., Rojo-Pérez F., Petidier R., Rodríguez-Rodríguez V., Forjaz M.J., Prieto-Flores M.E., de Pedro Cuesta J. (2009). Validación de la Escala de Independencia Funcional. [Validity of Functional Independence Measure]. Gac. Sanit..

[12] Cid-Ruzafa J., Damián-Moreno J. (1997). Disability evaluation: Barthel’s index. Rev. Esp. Salud Publica.

[13] Alvarez Solar M., de Alaiz Rojo A.T., Brun Gurpegui E., Cabañeros Vicente J.J., Calzón Frechoso M., Cosío Rodríguez I., García López P., García-Cañedo Fernández R., Pardo González I., Suárez-González A. (1992). Functional capacity of patients over 65 according to the Katz index. Reliability of the method. Aten. Primaria.

[14] Suárez S. (2012). Incidencia y pronósticos general y funcional de fracturas de cadera en población anciana. [Incidence and general and functional forecasts of hip fracture in elderly]. Canar. Médica Quirúrgica.

[15] Kristensen M.T., Kehlet H. (2012). Most patients regain prefracture basic mobility after hip fracture surgery in a fast-track programme. Dan Med. J..

[16] Kristensen M.T., Kehlet H. (2018). The basic mobility status upon acute hospital discharge is an independent risk factor for mortality up to 5 years after hip fracture surgery: Survival rates of 444 pre-fracture ambulatory patients evaluated with the Cumulated Ambulation Score. Acta Orthop..

[17] Fitzgerald M., Blake C., Askin D., Quinlan J., Coughlan T., Cunningham C. (2018). Mobility one week after a hip fracture—Can it be predicted?. Int. J. Orthop. Trauma Nurs..

[18] Kristensen M., Bandholm T., Foss N., Ekdahl C., Kehlet H. (2008). High inter-tester reliability of the new mobility score in patients with hip fracture. J. Rehabil. Med..

[19] Ariza-Vega P., Lozano-Lozano M., Olmedo-Requena R., Martín-Martín L., Jiménez-Moleón J.J. (2017). Influence of Cognitive Impairment on Mobility Recovery of Patients with Hip Fracture. Am. J. Phys. Med. Rehabil..

[20] Asplin G., Carlsson G., Zidén L., Kjellby-Wendt G. (2017). Early coordinated rehabilitation in acute phase after hip fracture—A model for increased patient participation. BMC Geriatr..

[21] Maranesi E., Riccardi G.R., Lattanzio F., Di Rosa M., Luzi R., Casoni E., Rinaldi N., Baldoni R., Di Donna V., Bevilacqua R. (2020). Randomised controlled trial assessing the effect of a technology-assisted gait and balance training on mobility in older people after hip fracture: Study protocol. BMJ Open.

[22] Schnell S., Friedman S.M., Mendelson D.A., Bingham K.W., Kates S.L. (2010). The 1-Year Mortality of Patients Treated in a Hip Fracture Program for Elders. Geriatr. Orthop. Surg. Rehabil..

[23] National Office of Clinical Audit (2018). Irish Hip Fracture Database National Report 2017.

[24] Azagra R., López-Expósito F., Martin-Sánchez J.C., Aguyé-Batista A., Gabriel-Escoda P., Zwart M., Díaz-Herrera M.A., Pujol-Salud J., Iglesias Martínez M., Puchol-Ruiz N. (2015). Incidence of hip fracture in Spain (1997–2010). Med. Clín..

[25] Ramada-Rodilla J.M., Serra-Pujadas C., Delclós-Clanchet G.L. (2013). Cross-cultural adaptation and health questionnaires validation: Revision and methodological recommendations. Salud Publica Mex..

[26] Kristensen M.T., Nielsen A.Ø., Topp U.M., Holmehave-Brandt J., Petterson C.F., Gebuhr P. (2018). Development and psychometric properties of the Basic Amputee Mobility Score for use in patients with a major lower extremity amputation: Basic Amputee Mobility Score. Geriatr. Gerontol. Int..

[27] Martínez De La Iglesia J., Herrero R.D., Vilches M.C.O., Taberné C.A., Colomer C.A., Luque R.L. (2001). Cross-cultural adaptation and validation of Pfeiffer’s test (Short Portable Mental Status Questionnaire [SPMSQ]) to screen cognitive impairment in general population aged 65 or older. Med. Clin..

[28] Pfeiffer E. (1975). A Short Portable Mental Status Questionnaire for the Assessment of Organic Brain Deficit in Elderly Patients. J. Am. Geriatr. Soc..

[29] Ariza-Vega P., Mora-Traverso M., Ortiz-Piña M., Ashe M.C., Kristensen M.T. (2019). Translation, inter-rater reliability, agreement, and internal consistency of the Spanish version of the cumulated ambulation score in patients after hip fracture. Disabil. Rehabil..

[30] Nunnally J.C. (1978). Psychometric Theory.

[31] Hopkins W.G. (2000). Measures of Reliability in Sports Medicine and Science. Sport. Med..

[32] Gliem J.A., Gliem R.R. Calculating, Interpreting, and Reporting Cronbach’s Alpha Reliability Coefficient for Likert-Type Scales. Proceedings of the Midwest Research-to-Practice Conference in Adult, Continuing, and Community Education.

[33] Tavakol M., Dennick R. (2011). Making sense of Cronbach’s alpha. Int. J. Med. Educ..

[34] Altman D.G., Bland J.M. (1983). Measurement in Medicine: The Analysis of Method Comparison Studies. Stat.

[35] Giavarina D. (2015). Understanding Bland Altman analysis. Biochem. Medica.

[36] Bland J.M., Altman D.G. (1997). Statistics notes: Cronbach’s alpha. BMJ.

[37] Mondragón Barrera M.A. (2014). Uso de la correlacion de Spearman en un estudio de intervención en fisioterapia. [Use of the correlation Spearman in a study of intervention in physiotherapy]. Mov. Científico.

[38] Sheehan K.J., Fitzgerald L., Hatherley S., Potter C., Ayis S., Martin F.C., Gregson C.L., Cameron I.D., Beaupre L.A., Wyatt D. (2019). Inequity in rehabilitation interventions after hip fracture: A systematic review. Age Ageing.

[39] Hulsbæk S., Larsen R.F., Troelsen A. (2015). Predictors of not regaining basic mobility after hip fracture surgery. Disabil. Rehabil..

[40] Ranhoff A.H., Holvik K., Martinsen M.I., Domaas K., Solheim L.F. (2010). Older hip fracture patients: Three groups with different needs. BMC Geriatr..

[41] Bodilsen A.C., Pedersen M.M., Petersen J., Beyer N., Andersen O., Smith L.L., Kehlet H., Bandholm T. (2013). Acute hospitalization of the older patient: Changes in muscle strength and functional performance during hospitalization and 30 days after discharge. Am. J. Phys. Med. Rehabil..

[42] Overgaard J., Kristensen M.T. (2013). Feasibility of progressive strength training shortly after hip fracture surgery. World J. Orthop..

[43] Steihaug O.M., Gjesdal C.G., Bogen B., Kristoffersen M.H., Lien G., Hufthammer K.O., Ranhoff A.H. (2018). Does sarcopenia predict change in mobility after hip fracture? A multicenter observational study with one-year follow-up. BMC Geriatr..

